# Improving the Transduction Efficiency and Antitumor Effect of Conditionally Replicative Adenovirus by Application of 6-Cyclohexyl Methyl-β-D-maltoside

**DOI:** 10.3390/molecules28020528

**Published:** 2023-01-05

**Authors:** Wenjing Lu, Yaping Fang, Xue Meng, Xiaoli Wang, Wenbo Liu, Mengdong Liu, Ping Zhang

**Affiliations:** Beijing International Science and Technology Cooperation Base of Antivirus Drug, Faculty of Environment and Life, Beijing University of Technology, Beijing 100124, China

**Keywords:** 6-cyclohexyl methyl-β-D-maltoside, transduction efficiency, conditionally replicative adenovirus, lethal effect, gene therapy

## Abstract

As a tumor-targeting oncolytic adenovirus (Ad), conditionally replicating adenovirus (CRAd) can access the cell interior by binding to coxsackievirus-Ad receptors (CARs) and specifically replicate and destroy cancer cells without lethal effects on normal cells. The transduction efficiency of CRAd is highly dependent on the number of CARs on the cell membrane. However, not all tumor cells highly express CARs; therefore, improving the transduction efficiency of CRAd is beneficial for improving its antitumor effect. In this study, 6-cyclohexyl methyl-β-D-maltoside (6-β-D), as maltoside transfection agent, showed several advantages, including high transfection efficiency, low toxicity, and potential for intensive use and easy operation. With pretreatment of cancer cells with low concentration of 6-β-D (≤5 μg/mL), the transduction efficiency of “model” Ad (eGFP-Ad) was improved 18-fold compared to eGFP-Ad alone. 6-β-D improved the antitumor effect of CRAd while being safe for normal cells, in which treatment with 6-β-D helped the lethal effects of CRAd at a multiplicity-of-infection ratio of 10 (MOI 10) achieve the oncolytic outcomes of MOI 50. This means that if CRAd is combined with 6-β-D, the amount of CRAd used in clinical practice could be greatly reduced without diminishing its curative effect or exposing patients to the potential side effects of high-titer CRAd. Finally, the underlying mechanism of antitumor effect of CRAd + 6-β-D was primarily investigated, and we found that 6-β-D increased the virus’s replication in cancer cells at the early stage of infection and activated the apoptosis signaling pathway at the late stage of the cell cycle. This research will provide an effective technical reference for further improving Ad-mediated cancer gene therapy in clinical practice.

## 1. Introduction

Conditionally replicating adenovirus (CRAd) is a tumor-targeting oncolytic adenovirus used in cancer gene therapy [1,2,3,4]. As a carrier of tumor-specific survivin promoter, CRAd can selectively replicate in cancer cells under the effect of survivin and lyse those cells without lethal effect on normal cells [5,6,7,8,9]. The adenoviruses mainly attach to coxsackie/Ad receptors (CARs) on cell surfaces to access into cells. However, CARs are lacking on the membrane of some kinds of tumor tissues and cells, which leads to a lower Ad transduction efficiency [2,10,11]. Therefore, it is important to efficiently transfer exogenous genes into cancer cells to improve therapeutic outcomes in cancer gene therapy [12,13,14].

Transfection agents with high efficiency and low toxicity have been considered a promising approach to overcoming this serious obstacle. Currently, commonly used transfection reagents include calcium phosphate, nanomaterials, liposomes, and non-liposomal agents [3,15,16,17,18,19]. The glycoside agent 6-cyclohexyl methyl-β-D-maltoside (6-β-D) is a bipolar molecule with a hydrophilic polar group and a hydrophobic nonpolar group. It has been reported that 6-β-D can insert into the cell membrane, break down membrane lipids, and release membrane proteins without disturbing the structural stability of the membrane proteins [20,21,22], thereby altering cell membrane permeability and mediating exogenous substances into cells. Theoretically, as a non-liposomal agent, 6-β-D shows several advantages in gene- and drug-delivery systems, including high transduction efficiency, low toxicity, intensive applications, and easy operation. However, up to now, research on the use of 6-β-D as a new gene delivery carrier in Ad-mediated gene therapies has been limited.

In this study, the appropriate doses of 6-β-D with high transduction efficiency and low toxicity were determined. Based on the safe dose, we measured the lethal effects of CRAd with and without 6-β-D on different human cancer cells. In addition, the antitumor mechanism of CRAd was explored. The results showed that 6-β-D at very low concentration (several ppm) could significantly improve the transduction efficiency of Ad into cells. CRAd had a broad-spectrum killing effect on different types of cancer cells, while safe to human normal cells. The lethal effect of CRAd showed positive relationships with multiplicity-of-infection ratio (MOI) and treatment time. CRAd replication significantly increased in 6-β-D-pretreated human cancer cells. When combined with 6-β-D, CRAd at MOI 10 achieved the killing effect of MOI 50 on cancer cells. It is suggested that the viral titer could be greatly reduced in clinical practice, which could free patients from the potential adverse effects of high-titer adenoviruses. This study will provide technical support and a theoretical basis for improving the clinical application of Ad in cancer gene therapy.

## 2. Results and Discussion

### 2.1. Conditional Replication Adenoviruses Can Kill Cancer Cells Specifically

**Figure 1:**
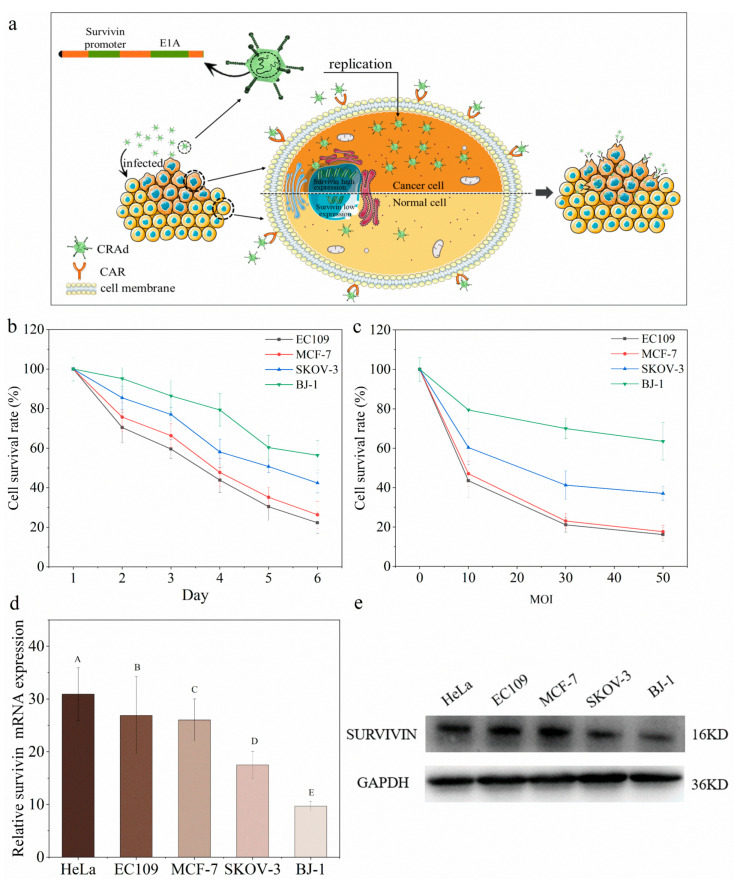
CRAd can replicate and package the offspring adenoviruses specifically in cancer cells. (**a**) Proposed antitumor mechanism of CRAd; (**b**) Antitumor effects of CRAd (MOI 10) on different cells for different durations (in days); (**c**) Antitumor effects of CRAd at varying MOI ratios on the 4th day; (**d**) Survivin mRNA expression in various cell lines; (**e**) Survivin protein in various cells, as detected by Western blot. Quantitative analysis of survivin protein could been seen in Supplementary Materials. Note: The different letters indicate that differences reached extremely significant levels (*p* < 0.001).

As shown in Figure 1a, CRAds enter into cells by recognizing the glycoprotein CAR receptors on the cell surface. CRAd contains a specific survivin promoter before the E1A gene. It has been widely shown that the survivin gene is highly and specifically expresses in many human tumors, but absent in normal adult differentiated cells [6,7,8,9]. The incorporation of a survivin promoter leads to CRAd’s specific replication in human cancer cells, and eventually kills them to release offspring viruses to further infect neighboring cancer cells. Therefore, the Ad vector with a survivin promoter has potential to target human cancer cells.

CRAds had a broad-spectrum killing effect on different types of cancer cells, while being safe for human normal cells. There is a positive antitumor correlation with the multiplicity of infection or duration of treatment (Figure 1b,c). However, different types of cancer cells showed different sensitivities to CRAds. Specifically, the most efficient oncolytic effects occurred on esophageal cancer cells EC109 and breast cancer cells MCF-7. As for the human normal skin fibroblast cells, the survival rate of BJ-1 cells was 85.43 ± 3.56% at MOI 10, indicating that CRAd was safe for human normal cells. 

The CRAds were more effective in the treatment of esophageal and breast cancers, but less so in ovarian cancer, because only 60% of SKOV-3 cells were killed even as the multiplicity of infection reached 50. This may be due to the membrane of ovarian cancer cells lacking CAR receptors [2,10,11], thus hindering adenoviruses entering the SKOV-3 cells through the CAR receptors. Moreover, survivin gene expression was lower in ovarian cancer cells SKOV-3 and normal BJ-1 cells than that in breast cancer cells MCF-7 and esophageal cancer cells EC109 (Figure 1d,e). These above results collectively explain the varying oncolytic effects of CRAds on different cancer cells.

### 2.2. High Transduction Efficiency and Low Cytotoxicity of 6-β-D

The killing effect of CRAd on cancer cells can be improved by increasing the viral titer in vitro. However, this is not desirable in vivo because of the potential side effects in practice. It is important to allow more CRAds to invade cancer cells and replicate and package more offspring viruses to achieve better curative effects. Therefore, using transfection reagents to improve the transduction efficiency of CRAd was considered.

**Figure 2:**
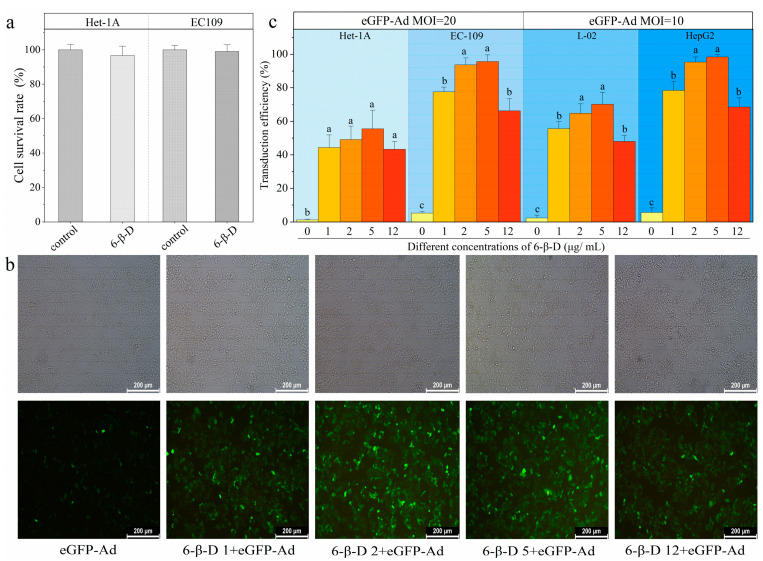
When eGFP was used as a reporter gene, 6-β-D showed low cytotoxicity and high transduction efficiency in different human cell lines. (**a**) Cytotoxicity of 6-β-D on Het-1A and EC109 cells; (**b**) 6-β-D promoted eGFP expression in EC109 cells; (**c**) Transduction efficiency of eGFP-Ad was affected by different concentrations of 6-β-D. Note: The same letter means no significant difference (*p* > 0.05), while different letters indicate that differences reached significant levels (*p* < 0.05).

In this study, a glycoside material, 6-cyclohexyl methyl-β-D-maltoside (6-β-D), was selected and used to explore its transduction efficiency. The 6-β-D is a bipolar molecule with a hydrophilic polar group and a hydrophobic non-polar group that can help foreign substances invade target cells by changing the permeability of cell membranes [20,21,22]. As shown in Figure 2a, the cell viability showed no difference between 6-β-D-treated (12 μg/mL) and untreated cells, indicating 6-β-D at this concentration exhibited an unobvious inhibitory effect on cell growth (*p* > 0.05). 

We adopted the Ad with enhanced GFP expression (eGFP-Ad) as a “reporter” to rapidly and quantitatively evaluate the transduction effect of 6-β-D. By directly observing the amount of lightened GFP^+^ cells, we found that the transduction rate of Ad alone was only 5%, while the 6-β-D treatment showed remarkably more GFP expression (Figure 2b). It indicated that more eGFP-Ad invaded cells with application of 6-β-D. There was a correlation between the 6-β-D concentration and the transduction efficiency. The most efficient concentration of 6-β-D was 2~5 μg/mL in vitro cellular system, at which >95% of EC109 and HepG2 cells were successfully transfected by eGFP-Ad (Figure 2b,c). The transduction efficiency was improved 18-fold with application of 6-β-D (Figure 2c). 

In addition, comparing the transduction rates between cancer cells and normal cells, eGFP-Ad was more efficient in infecting cancer cells than normal cells under the action of 6-β-D. In Figure 2c, the transduction rates of cancer cells HepG2 and EC109 were 98.37 ± 1.56% and 95. 67 ± 4.04%, respectively, while they were 70.27 ± 7.16% and 55.60 ± 11.02% in normal cells L02 and Het-1A, respectively. Moreover, the 6-β-D was safe for cultured cells at a concentration of below 5 μg/mL. This meant that it was unnecessary to remove the 6-β-D solution after pretreating cells at low concentrations (<5 μg/mL). Thus, the experimental steps can be simplified, making the operation more convenient. Collectively, the results proved that 6-β-D showed more advantages as a transfection reagent, including high efficiency, low toxicity, low cost, and easy operation. 

### 2.3. Application of 6-β-D Improved the Oncolytic Effect of CRAd

**Figure 3:**
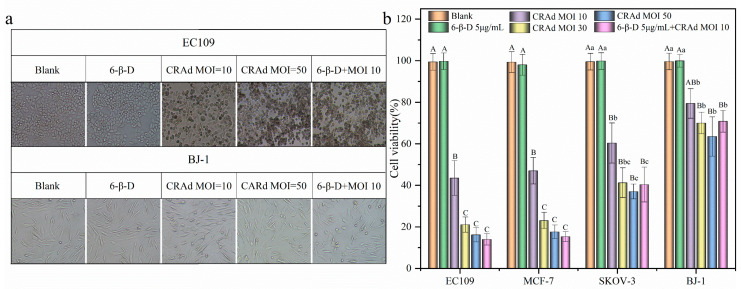
Application of 6-β-D improved the oncolytic effect of CRAd. (**a**) Comparison of cytopathic effects on esophageal cancer cells EC109 vs. human normal skin fibroblast cells BJ-1 after infection with different titers of CRAd (×200); (**b**) The synergistic killing effect of CRAd (MOI 10) + 6-β-D achieved outcomes of higher MOI 50 on various cancer cell lines. Note: Different letters indicate significant difference in pairwise comparison, while the same letter means no significant difference. The difference was significant in lowercase letters (*p* < 0.05) and extremely significant in uppercase letters (*p* < 0.01).

Compared with normal cells BJ-1, CRAd had a significant killing effect on cancer cells (*p* < 0.01, Figure 1b), and the killing effect was positively correlated with multiplicity of infection (Figure 1c). As shown in Figure 3, when CRAd of MOI 10 infected cancer cells, on the fourth day, the survival rates of EC109 and MCF-7 cells were 43.49 ± 3.26% and 47.04 ± 2.53%, respectively. As MOI reached 50, cell survival rate decreased to 15% (Figure 3a). After 6-β-D pretreatment, CRAd at MOI 10 could further improve the killing effect on cancer cells. The lethal rates were as high as 85.33 ± 1.64% and 84.68 ± 2.74% in EC109 and MCF-7 cells, respectively, which were significantly different from that of CRAd alone (*p* < 0.01, Figure 3b).

The oncolytic effect of CRAd (MOI 10) + 6-β-D was similar to that of CRAd with high viral titer (MOI 50) (*p* > 0.05, Figure 3). In these treated groups, almost all the cancer cells were killed and floated, and the few remaining cells were weak and round in shape (Figure 3a). This meant the viral titer could be reduced by combining 6-β-D with CRAd in clinical practice. It can be used as an auxiliary reagent to obtain higher gene-transduction efficiency, thus reducing the amount of CRAd in Ad-based gene therapy.

However, for normal cells BJ-1, most of them still adhered to the culture flask, and the cellular morphology was intact without obvious damage (Figure 3a). This result further proved that CRAd selectively killed cancer cells and had a lower attacking effect on normal cells. 

### 2.4. Combining 6-β-D with CRAd Increased Virus Replication in Cancer Cells

E1A is an early-expressed gene related to adenovirus replication [5,23,24]. In this experiment, we detected the expression of E1A to evaluate the amount of CRAds entering cells after drug treatment. First, cells were treated with 6-β-D for about 20 min and then infected with CRAd of MOI 10. After 4 h, the cells were flushed with PBS three times to remove the superfluous viruses and replaced with fresh medium to cultivate for another 24 h. Thereafter, the DNA copy number and mRNA expression of E1A were detected. 

**Figure 4:**
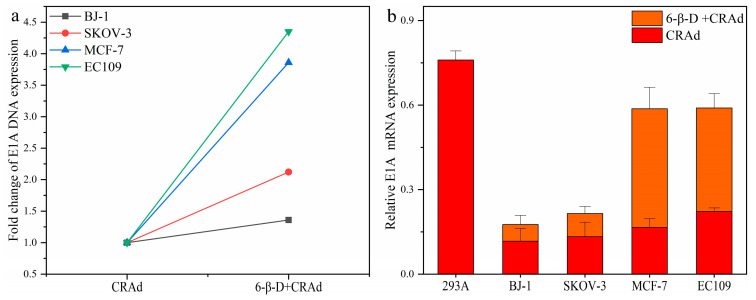
Combination of 6-β-D with CRAd increased viral replication in various cancer cell lines. (**a**) 6-β-D promoted CRAds invasion into cancer cells; (**b**) As detected by Real time-PCR, 6-β-D increased CRAd–E1A mRNA expression in cancer cells.

As shown in Figure 4a,b, E1A gene expression in 6-β-D + CRAd-treated cells increased significantly over that in 6-β-D-untreated cells; especially in EC109 and MCF-7 cells, the DNA copy number increased nearly four-fold (Figure 4a). This indicates that CRAd invasion of cancer cells was improved by 6-β-D treatment. Meanwhile, CRAd-E1A mRNA expression was significantly higher in EC109 and MCF-7 cells than in BJ-1 cells (Figure 4b). The higher expression of E1A was consistent with the killing effect of CRAd. 

Furthermore, the TCID_50_ method was applied to detect CRAd replication and amplification in EC109 and SKOV-3 cells with and without 6-β-D. It was reported that most adenoviruses could enter cells within 3 or 4 h of infection. During this period, the intracellular viruses had not yet packaged the offspring viruses. Therefore, the viral titer detected at 3 h reflected the amount of CRAds entering cells. After 96 h of infection, CRAds replicated numerous progeny viruses. The results collected at this stage reflected the replication ability of CRAds. Therefore, the viral titers were analyzed after 3 h and 96 h infection. 

As shown in Table 1, 6-β-D treatment facilitated more CRAds entering cancer cells at the initial stage of infection. The viral titer had increased 1.5-fold and 1.58-fold in SKOV-3 and EC109 cells, respectively, while 96 h later, the CRAds proliferated more rapidly in EC109 cells than in SKOV-3 cells. The 6-β-D-treated group was 4.9-fold of the untreated one. This explains why the killing effect of CRAds on EC109 cells was better than that on SKOV-3 cells.

In brief, the above results indicated that intracellular CRAds can exert a strong cytopathic effect on cancer cells with high E1A and survivin expression. The more oncolytic CRAds entered cells, the more CRAds replicated in cancer cells, and thus more significant killing effects were exhibited on cancer cells.

**Figure 5:**
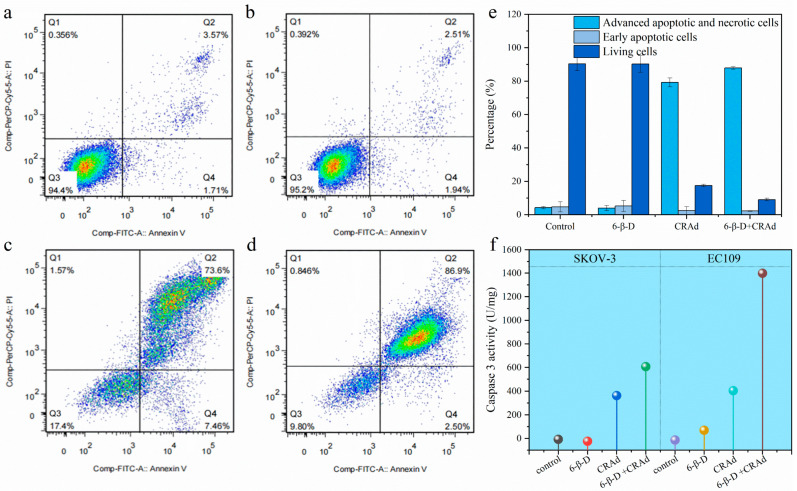
Effects of CRAd on Caspase-3 activity and cellular apoptosis. CRAd-induced cellular apoptosis detected by flow cytometry. (**a**) Control: EC109 cells; (**b**) 6-β-D-treated EC109 cells; (**c**) CRAd-treated EC109 cells; (**d**) 6-β-D- and CRAd–treated EC109 cells; (**e**) Quantitative analysis of cellular apoptosis; (**f**) CRAd stimulated Caspase-3 activity in EC109 and SKOV-3 cells.

According to the flow cytometry (FCM) results, the proportions of apoptotic and necrotic cells in CRAd-treated groups were significantly higher than those in 6-β-D-treated and untreated groups (Figure 5a–e). Moreover, there was no significant difference between 6-β-D-treated and untreated groups, indicating the safety of 6-β-D to human cells (Figure 5a–e). In Figure 5c,d, the cellular apoptotic rate increased from 73.6% to 86.9%, indicating that 6-β-D promoted the killing effect of CRAd at cell-safe doses.

Caspase-3 is an initial protein of the apoptosis pathway. In Figure 5f, Caspase-3 activity in both CRAd-treated EC109 and SKOV-3 cells was significantly higher than that of untreated cells. This result suggested that CRAd treatment stimulated Caspase-3 activity and initiated the cellular apoptosis pathway. Furthermore, Caspase-3 activity in EC109 cells was generally greater than that in SKOV-3 cells under 6-β-D + CRAd treatment (Figure 5f), verifying the better killing effect of CRAd on esophageal cancer cells. These data corresponded to the previous results.

Collectively, we speculate that CRAd may exert its lethal effect on cancer cells through two different mechanisms. First, at the early stage of infection, it mainly does so via specific replication in cancer cells to produce more progeny adenoviruses to cause cytolysis. Second, CRAd replication stimulates Caspase-3 activity, which triggers the apoptosis signaling pathway at the late stage of the cell cycle.

## 3. Materials and Methods

### 3.1. Cell Lines and Drugs

The human cancer cells (including esophageal cancer cell EC109, liver cancer cell HepG2, breast cancer cell MCF-7, ovarian cancer cell SKOV-3, and cervical cancer cell HeLa), human normal esophageal cell (Het-1A), and human normal skin fibroblast (BJ-1) cell lines were obtained from the National Collection of Authenticated Cell Cultures (Shanghai, China). The human normal liver cell line (L-02) was collected from the China Center for Type Culture Collection (Wuhan, China). 6-β-D was purchased from Sigma-Aldrich (St. Louis, MO, USA). The CCK-8 kit and bicinchoninic acid (BCA) kit were acquired from Beyotime Institute of Biotechnology (Shanghai, China). An AceQ quantitative polymerase chain reaction (qPCR) kit was obtained from Vazyme Biotech Co., Ltd. (Nanjing, China). TRIzol LS Regent, which was used for extraction of total ribonucleic acid (RNA), was purchased from Tiangen Biotech Co., Ltd. (Beijing, China). CRAd and eGFP-Ad were constructed and preserved in our laboratory in accordance with previously published methods [5]. Briefly, CRAd with strong cancer-dependent proliferation was created by introducing the cancer-specific survivin promoter to regulate adenoviral E1A gene expression, and the E1B gene was deleted from the Ad.

### 3.2. Cytotoxicity Assay

The cellular toxicity of 6-β-D was detected using the CCK-8 method [25,26]. First, EC109 and Het-1A cells were seeded in a 96-well plate at a density of 8 × 10^3^/well and cultured at 37 °C with 5% CO_2_ for 24 h. Then, each well was incubated with 100 μL 6-β-D (max. 12 μg/mL) for about 20 min. The regent was discarded and replaced with fresh Dulbecco’s Modified Eagle’s medium (DMEM), and cells were cultivated for another 48 h. Finally, the cytotoxicity test was conducted according to the CCK-8 protocol. Optical density (OD) was measured at 450 nm using a Multiskan Sky microplate spectrophotometer (Thermo Fisher Scientific, Waltham, MA, USA). The cell viability was calculated according to Equation (1), as follows. The experiment was repeated 3 times.

Cell survival rate (%) = (OD_treatment_ − OD_blank_)/(OD_control_ − OD_blank_) × 100% (1)

### 3.3. Transduction Efficiency

We adopted a “model” Ad (eGFP-Ad) to evaluate how well 6-β-D improved the transduction efficiency. The concentrations of 6-β-D solution were 1, 2, 5, and 12 μg/mL. Before virus infection, 100 μL of 6-β-D solution at different concentrations was separately added to each well. After 20 min, HepG2 and L-02 cells were transducted with MOI 10 and EC109 and Het-1A cells with MOI 20 of eGFP-Ad for 4 h. Then, the drug solution was replaced with 500 μL fresh DMEM medium, and cells were cultivated for another 48 h. Next, the GFP expression was observed under a fluorescence microscope, counting GFP^+^-cells and comparing counts between the 6-β-D-treated and -untreated groups. The transduction efficiency was calculated as the ratio of the percentage of GFP-expressing cells to that of whole cells under the observation field. For each sample, five observation fields were randomly selected for data collection, and each experiment was repeated 3 times.

### 3.4. Survival Inhibition Effect Assay

According to the selected dosage of 6-β-D used in the transduction efficiency assay, the synergistic effect of 6-β-D and CRAd in killing cancerous cells was analyzed using the CCK-8 method. Three cancer cell lines (EC109, MCF-7, and SKOV-3 cells) and one human normal cell line (BJ-1) were selected and seeded in a 96-well plate at a density of 8 × 10^3^/well and cultured for 24 h. For each cell line, four treatment groups were established: the CRAd (MOI 10, 30, 50)-treated group, 6-β-D (5 μg/mL)-treated group, 6-β-D (5 μg/mL) + CRAd-treated (MOI 10) group, and blank control group. After treatment for 4 days, cell survival rate was calculated according to Equation (1).

### 3.5. RNA Preparation and RT-PCR

Total RNA was prepared using TRIzol LS Regents (DP430, Tiangen, Beijing, China). Reverse transcription was performed using Hiscipt^®^IIQ Select RT SuperMix for qPCR (+gDNA wiper) (Vazyme Biotech Co., Ltd., Nanjing, China). The E1A and survivin gene expressions were quantitatively analyzed using SYBR Green qRT-PCR premix (TaKaRa, Kyoto, Japan) (2^−ΔΔCt^ method) on a fluorescent quantitative PCR system (MX3000P, Stratagene, La Jolla, CA, USA). PCR amplification used an AceQ qPCR kit (Q111-02/03, Vazyme Biotech Co., Ltd., Nanjing, China) and started with an initial denaturation step at 95 °C for 5 min; followed by 40 cycles of 95 °C for 30 s, 58 °C for 30 s, and 72 °C for 30 s; and a final elongation step at 72 °C for 3 min. Primer sequences for E1A were F: 5′-TCCTCACCCTCTTCATCCTC-3′, R: 5′-GAACCACCTACCCTTCACGA-3′. Primer sequences for survivin were F: 5′-CAGCCCTTTCTCAAGGACCAC-3′, R: 5′-TTTCTCCGCAGTTTCCTCAAA-3′. Glyceraldehyde 3-phosphate dehydrogenase (GAPDH) was an internal reference gene and its primer sequences were F: 5′-TTCCGTGTTCCTACCCCCAA-3′, R: 5′-AGCCCAAGATGCCCTTCAG-3′. These gene primers were designed using Premier software version 5.0 and synthesized by Sangon Biotech (Shanghai, China).

### 3.6. Viral Titer Assay

The viral titer was tested using the median tissue culture infectious dose (TCID_50_) assay, which was calculated according to Karber’s formula.

### 3.7. Flow Cytometry (FCM)

FCM determination was performed on 6-β-D-treated (5 μg/mL), CRAd (MOI 50)-treated, 6-β-D (5 μg/mL) + CRAd (MOI 10)-treated, and untreated cells. After treatment for 72 h, cells were harvested and stained with Annexin-V-Fluorescein isothiocyanate (FITC) and propidium iodide (PI). Then, the cells were analyzed on a FACScan flow cytometer equipped with CellQuest software version 5.1 (BD Biosciences, Franklin Lakes, NJ, USA).

### 3.8. Caspase-3 Activity Analysis

Caspase-3 activity was analyzed in four treatment groups: 6-β-D (5 μg/mL) only, CRAd (MOI 10) only, 6-β-D (5 μg/mL) + CRAd (MOI 10), and blank control. For this assay, a Caspase-3 Activity Assay Kit (C1116, Beyotime Biotechnology, Shanghai, China) was used according to the manufacturer’s protocol.

### 3.9. Statistical Analysis

Results were expressed as mean ± standard deviation (SD) for continuous variables. One-way analysis of variance (ANOVA) of Bonferroni’s post hoc test was used to compare differences among various groups using GraphPad Prism software version 8.0 (GraphPad Software, Inc., San Diego, CA, USA). *p* < 0.05 was considered statistically significant, and *p* < 0.01 was extremely significant.

## 4. Conclusions

Conditionally replicating adenoviral vectors, carrying a specific survivin gene promoter, tend to propagate in cancer cells and eventually lyse cancer cells. In this study, 6-β-D as a new transduction agent showed several advantages, including high transduction efficiency, low toxicity, intensive application, cost effectiveness, and easy operation. With application of 6-β-D, the lethal effect of CRAd at MOI 10 improved significantly, achieving the oncolytic outcomes associated with the use of a higher titer (MOI 50). This meant that by using 6-β-D + CRAd, clinicians could greatly reduce the amount of CRAd without reducing its curative effect, thereby sparing patients from most of CRAd’s potential side-effects caused by higher viral titer. Finally, the mechanism underlying the antitumor effect of CRAd was primarily investigated. This study will increase the feasibility of reducing viral titer to improve antitumor effects through the use of a safe transfection agent in adenovirus-based gene therapy. These insights may prove to be a timely opportunity for the application of CRAd in clinical treatment of cancers.

## Figures and Tables

**Table 1:** Viral titer of CRAd detected by TCID_50_.

Time	Treatment	Fold (Titer/PFU/mL)	
		SKOV-3	EC109
**3 h**	CRAd	1 (10^5.3^)	1 (10^5.5^)
	6-β-D + CRAd	1.50 (10^5.5^)	1.58 (10^5.7^)
**96 h**	CRAd	1 (10^9.5^)	1 (10^10.4^)
	6-β-D + CRAd	1.86 (10^10^)	4.90 (10^11.2^)

## Data Availability

Not applicable.

## Supplementary Materials

The following supporting information can be downloaded at: https://www.mdpi.com/article/10.3390/molecules28020528/s1, Figure S1: Quantitative analysis of survivin protein (Figure 1e); Figure S2: Survivin protein in various cells (HeLa, EC109, MCF-7, SKOV-3, BJ-1), as detected by Western blot (Figure 1e); Figure S3: GAPDH protein in various cells (HeLa, EC109, MCF-7, SKOV-3, BJ-1), as detected by Western blot (Figure 1e).
