# Supplementary material for: Improving the Transduction Efficiency and Antitumor Effect of Conditionally Replicative Adenovirus by Application of 6-Cyclohexyl Methyl-β-D-maltoside
Source: Molecules. 2023 Jan 5;28(2):528. doi: 10.3390/molecules28020528 (PMC9862058; doi:10.3390/molecules28020528)
Supplement: Supplementary file 1 [file molecules-28-00528-s001.zip › molecules-2071396-supplementary.pdf]

Supplementary Material

Improving the Transduction Efficiency and Antitumor Effect of Conditionally Replicative Adenovirus by Application of 6-cyclohexyl Methyl-β-D-maltoside

Wenjing Lu, Yaping Fang, Xue Meng, Xiaoli Wang, Wenbo Liu, Mengdong Liu, Ping Zhang *

Beijing International Science and Technology Cooperation Base of Antivirus Drug, Faculty of Environment and Life, Beijing University of Technology, Beijing, 100124, P. R. China

* Corresponding author: zplife@bjut.edu.cn (P. Zhang)

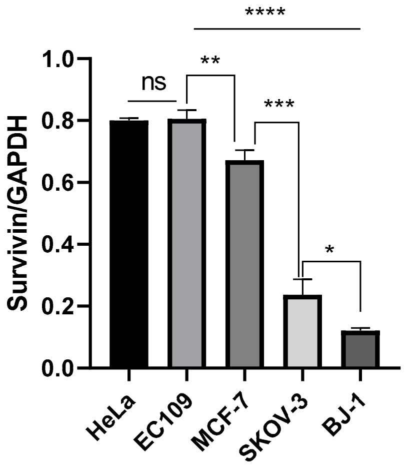

Supplementary Figure S1. Quantification of Western blot. Note：*P < 0.05, **P < 0.01, ***P < 0.005 and ****P < 0.001. (Figure 1e).

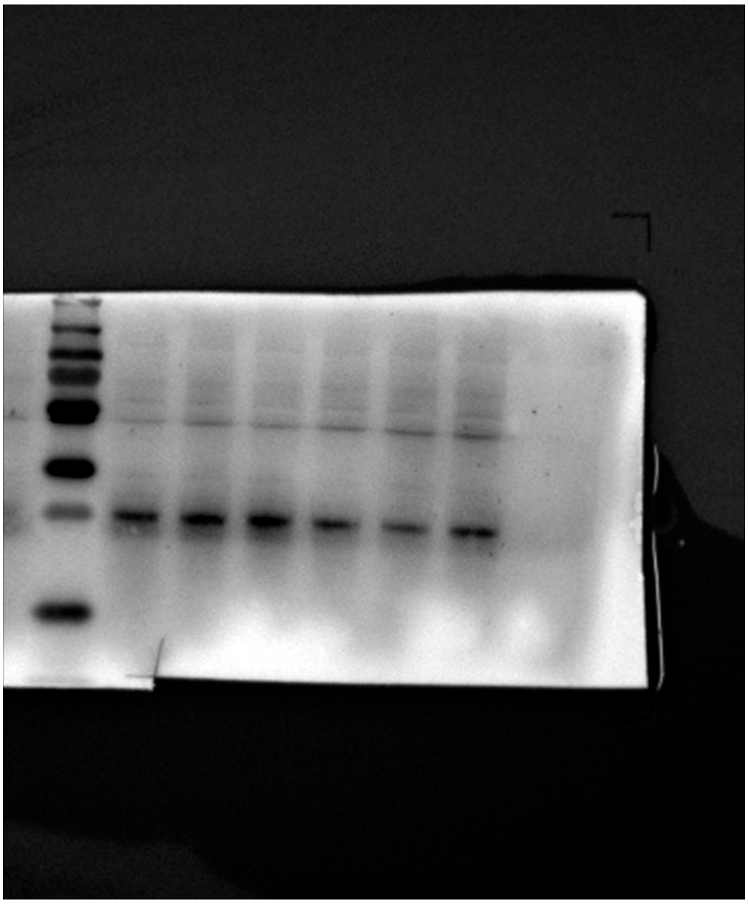

Supplementary Figure S2. Survivin protein in various cells (HeLa, EC109, MCF-7, SKOV-3, BJ-1), as detected by Western blot (Figure 1e).

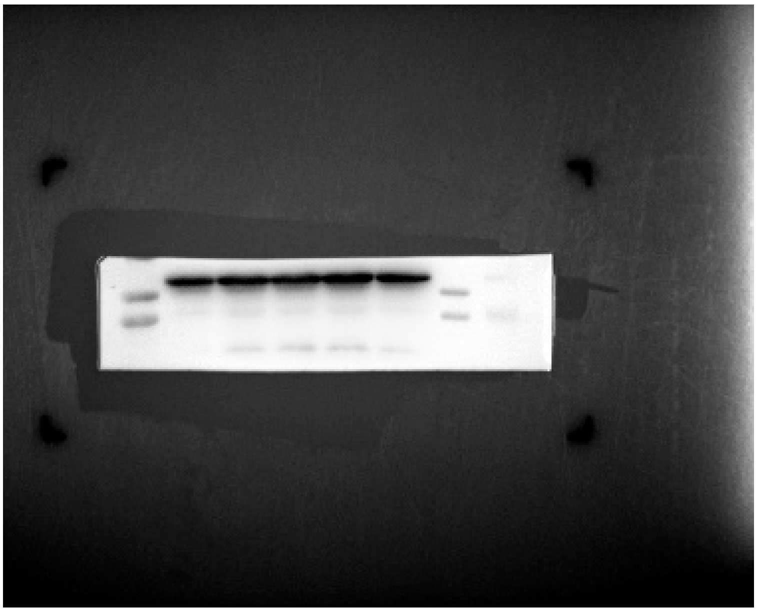

Supplementary Figure S3. GAPDH protein in various cells (HeLa, EC109, MCF-7, SKOV-3, BJ-1), as detected by Western blot (Figure 1e).
